# *QuickStats:* Age-Adjusted Female Breast Cancer Death Rates,* by State — United States, 2024

**DOI:** 10.15585/mmwr.mm7540a3

**Published:** 2026-10-15

**Figure:**
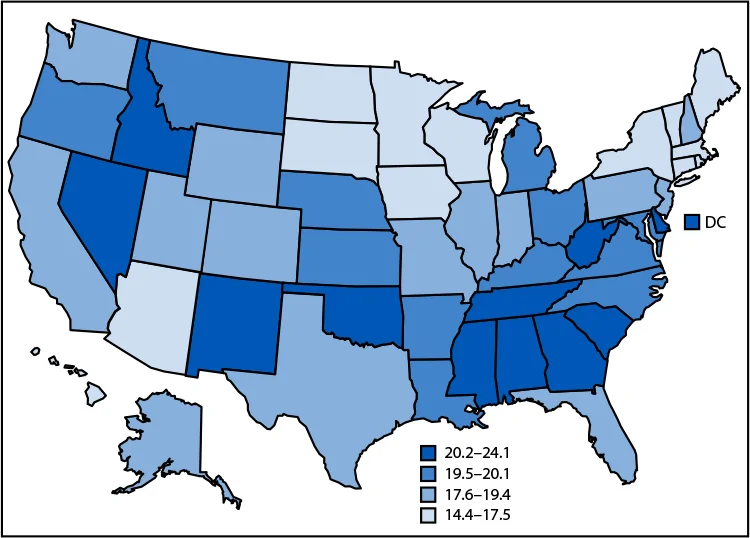
In 2024, the age-adjusted rate of female breast cancer deaths in the United States was 18.7 per 100,000 females. DC had the highest breast cancer death rate (24.1), and Rhode Island had the lowest (14.4).

**Supplementary Table:** Age-Adjusted Female Breast Cancer Death Rates, by State — United States, 2024

**Source:** National Center for Health Statistics, National Vital Statistics System, Mortality 2024. Underlying Cause of Death, 2018–2024, Single Race Request

